# A brief CBT intervention for depersonalisation/derealisation in psychosis: study protocol for a feasibility randomised controlled trial

**DOI:** 10.1186/s40814-016-0086-7

**Published:** 2016-08-11

**Authors:** Simone Farrelly, Emmanuelle Peters, Matilda Azis, Anthony David, Elaine C Hunter

**Affiliations:** 1 Department of Psychology, Institute of Psychiatry, Psychology and Neuroscience, King’s College London, London, UK; 2 NIHR Biomedical Research Centre for Mental Health, South London and Maudsley NHS Foundation Trust, London, UK; 3 Department of Psychological Medicine, Institute of Psychiatry, Psychology and Neuroscience, King’s College London, London, UK; 4 Department of Psychosis Studies, Institute of Psychiatry, Psychology and Neuroscience, King’s College London, London, UK

**Keywords:** Psychosis, Depersonalisation, Derealisation, Cognitive behaviour therapy, Intervention, Feasibility trial

## Abstract

**Background:**

Depersonalisation is the experience of being detached or disconnected from one’s experience. Studies suggest that clinically significant levels of depersonalisation are common in individuals who have psychotic symptoms and are associated with increased impairment. However, to date, there have been no studies that have investigated an intervention designed to target clinically significant depersonalisation in such patient groups. This study aims to determine the feasibility and acceptability of a brief intervention targeting clinically significant depersonalisation in those who also have current psychotic symptoms.

**Methods/design:**

The feasibility of delivering six sessions of cognitive behavioural therapy for depersonalisation in psychosis patients will be evaluated using a single-blinded randomised controlled trial with a treatment as usual control condition. Participants will be assessed at baseline and then randomised to either the treatment or control arm. Participants randomised to the treatment arm will be offered six sessions of individual cognitive behavioural therapy delivered over a maximum of 10 weeks. Therapy will focus on an individualised shared formulation of depersonalisation experiences and behavioural, cognitive, emotional regulation and thinking process strategies to decrease distress associated with depersonalisation. Participants will be assessed again at a 10-week (post-randomisation) follow-up assessment. The primary outcomes of interest will be those assessing the feasibility and acceptability of the intervention including rates of referral, eligibility and acceptance to participate; attendance at therapy sessions and completion of homework tasks; satisfaction with the intervention; maintenance of blinding; and therapist competence. Secondary outcomes will be data on clinical outcome measures of depersonalisation and positive symptoms of psychosis, anxiety, depression and post-traumatic stress.

**Discussion:**

This study will determine the feasibility of delivering six sessions of cognitive behavioural therapy for individuals with current psychotic symptoms who also experience clinically significant levels of depersonalisation. The results will provide information to inform a larger randomised trial to assess intervention efficacy.

**Trial registration:**

ClinicalTrials.gov NCT02427542

## Background

Psychosis is a general term covering a range of psychiatric diagnoses such as schizophrenia, schizoaffective disorder and delusional disorder [1]. Psychotic symptoms include delusions, hallucinations, negative symptoms such as affective flattening and cognitive disturbances. Recent estimates suggest that four in every 1000 people in the United Kingdom (UK) have a diagnosis of a psychotic condition [2]. Alongside medication, current treatment guidelines [1] recommend psychological intervention using either cognitive behavioural therapy for psychosis (CBTp) or family interventions. Recent meta-analyses have shown some efficacy for CBTp [3]; however, prominent theorists and clinicians have called for further treatment innovations and understanding of the most efficacious treatment components [4–6]. One such approach is the ‘the causal-interventionalist’ approach [4], whereby a single hypothesised maintenance factor is targeted with CBT in order to reduce both this problem (e.g. worry, insomnia) with the secondary gain of improving psychotic, and other emotional, symptoms. Dissociation, and depersonalisation in particular, may be one such maintenance factor.

### Defining dissociation and depersonalisation

Dissociation is defined as ‘a disruption of and/or discontinuity in the normal integration of consciousness, memory, identity, emotion, perception, body representation, motor control and behaviour’ [7]. As such, it is an umbrella term that incorporates a spectrum of phenomena ranging from normal, everyday experiences such as absorption and divided attention to more distressing and functionally impairing experiences of clinical significance such as the psychiatric condition of depersonalisation disorder (DPD).

Historically, all types of dissociative phenomena have been viewed as part of a continuum, from ‘normative’ experiences to more pathological experiences. However, more recent theoretical reviews have suggested that dissociation may be best considered as comprising of two categories of phenomena: detachment and compartmentalisation [8], although these are not necessarily mutually exclusive. ‘Detachment’ concerns a person’s sense of separation from experience, including from their sense of self (i.e. depersonalisation) or from the external world (i.e. derealisation) [8, 9]. ‘Compartmentalisation’, on the other hand, is defined as a disruption in normally integrated functions that is not accessible to conscious control and includes dissociative amnesia and somatoform dissociation [8]. This study is primarily interested in the ‘detachment’ experiences of depersonalisation and derealisation (referred to as depersonalisation henceforth) in those with a diagnosis of a psychotic disorder.

### Prevalence estimates of depersonalisation

Depersonalisation symptoms are common in non-clinical and clinical populations, particularly amongst those with anxiety disorders where it is amongst the diagnostic criteria of panic disorder and post-traumatic stress disorder (PTSD) [10]. Epidemiological studies suggest lifetime prevalence rates of transient depersonalisation symptoms in the general population of between 26 and 74 % [11]. Community surveys examining the prevalence of DPD specifically, using standardised diagnostic criteria, suggest 1 month prevalence rates of between 1.2 and 2.4 %, and rates as high as 82.6 % have been reported comorbid with other psychiatric disorders [11].

### Dissociation and psychosis—common factors or pathway?

There has been an increasing interest in the presence of dissociative experiences in psychotic disorders, in part due to common aetiological factors of trauma and anxiety, and the potential for dissociation to play a mediating role in psychosis [12–14]. Dissociation has long been considered a psychological defence mechanism to protect the individual against intolerable events such as trauma (see [14]). It is also now well established that rates of trauma are high in psychosis [15]. Recent population-based studies in both the UK and the United States of America (USA) show high rates of lifetime experiences of sexual and physical abuse in those diagnosed with a psychotic disorder [16, 17]. Further, in a critical review of studies examining trauma in those with severe mental illnesses, rates of trauma exposure were between 49 and 100 % [18]. Considering the well-established link between trauma and dissociation and the high rates of trauma experiences in psychosis, it is therefore not surprising that dissociation is also common in psychosis [19].

Additionally, the cognitive model of DPD [20] has emphasised the role of anxiety and cognitive processes common to anxiety disorders, in the development and maintenance of DPD. Experimental research provides support for the influence of cognitive processes of attention, catastrophic appraisals and attribution biases in DPD [21]. Further, a longitudinal study of over 3000 participants in the UK found that childhood anxiety was a significant predictor of adult depersonalisation experiences [22]. Likewise, cognitive models of psychosis [23] emphasise the role of emotional processes, particularly anxiety, in the onset and maintenance cycle of psychotic symptoms [5, 24].

Considering the commonalities in maintenance processes of dissociation and psychosis, it is understandable that dissociation is also common in psychosis, and indeed, some authors have speculated that some psychotic symptoms, in particular auditory hallucinations, may actually be better understood as dissociative in nature [12, 14].

### Rates of depersonalisation in psychosis

Eleven studies were identified by a review of research investigating dissociation in psychosis [25]. The reviewers concluded that there was ‘solid empirical evidence’ that individuals with a diagnosis of schizophrenia have more frequent, and severe, dissociative experiences than non-clinical populations but less frequent and severe than those diagnosed with borderline personality disorder, PTSD, or dissociative identity disorder. Furthermore, they found a consistent association between experiences of dissociation and severity of delusions and hallucinations. However, there have been methodological flaws in many of these studies as they used a general measure of dissociation that includes aspects of amnesia, detachment and more ‘normative’ dissociation such as absorption in the same overall score. In this context, it is unclear precisely what aspects of dissociative experience were associated with psychosis.

More recently, a few studies have specifically investigated depersonalisation experiences in psychosis. For example, in a study of 147 inpatients with a diagnosis of schizophrenia, 17 % were found to meet threshold criteria for DPD. Similarly, studies examining depersonalisation symptoms in schizophrenia suggest that depersonalisation may be more common in those experiencing hallucinations, compared to those with delusions only, and when present are associated with more severe psychotic symptoms [26–28]. However, this is still an emerging field of enquiry perhaps in part due to the ‘diagnostic overshadowing’ of psychosis. In this context, there is only a limited understanding of both the rates of DPD and the phenomenology of depersonalisation in psychosis.

### Depersonalisation—an anomalous experience in psychosis?

One potential understanding of depersonalisation in psychosis is of depersonalisation symptoms as an anomalous experience that is interpreted in a distressing manner. In the cognitive behavioural therapy (CBT) model of DPD [20], catastrophic appraisals of transient depersonalisation experiences (such as of having damaged one’s brain or of incipient ‘madness’) serve to exacerbate and maintain symptoms. Similarly, in cognitive models of psychosis (e.g. [29, 30]), appraisals of *anomalous experiences* as personally relevant, threatening and/or attributed to an external cause are proposed to contribute to the development of psychotic symptoms. Depersonalisation symptoms could be considered a type of anomalous experience [23, 28], and although to the authors’ knowledge there are no empirical studies which have explored the appraisals of depersonalisation symptoms in those with psychosis, and in particular whether these symptoms might give rise to psychotic explanations in the absence of any alternative explanation for these phenomena, it may be that this could be a factor that precipitates, and/or maintains, psychosis.

A study of CBT for DPD [31] focused on generating less threatening explanations for the depersonalisation symptoms and reducing symptom-focused attention, avoidance and safety behaviours that were identified as maintaining factors. This study showed significant improvements in the experience of depersonalisation symptoms, depression and anxiety, with a third of participants no longer meeting threshold for DPD at the end of therapy. It is proposed that a similar approach to depersonalisation symptoms in psychosis might be effective in reducing the distress associated depersonalisation and may have a secondary impact of psychotic symptoms. This approach is in line with the ‘causal-interventionist’ approach [4] which has been proposed as the way forward for CBT for psychosis. To the authors’ knowledge, there are no published studies or trials of interventions for depersonalisation symptoms in psychosis.

### Summary and research questions

Depersonalisation symptoms appear to be prevalent in people diagnosed with psychotic disorders and when present, depersonalisation symptoms are linked with more severe psychotic symptoms. It is likely that negative appraisals of these anomalous experiences might act to precipitate, maintain and exacerbate psychotic symptoms. CBT has been found to be beneficial in patients with chronic DPD, and it would be valuable to ascertain if similar approaches to target depersonalisation symptoms in psychosis would be effective. This study aims to establish the feasibility of a brief CBT-based intervention for depersonalisation symptoms in people diagnosed with a psychotic disorder. The aim of the intervention would be to alter negative attributions and distress associated with depersonalisation experiences through psycho-education, learning coping strategies such as ‘grounding’, changing attentional biases and cognitive restructuring techniques to modify appraisals. It is proposed that through reducing distress, in particular, the maintenance cycle associated with depersonalisation will be altered and thus overall depersonalisation experiences reduced, with a possibility of reducing psychotic phenomena in addition.

In this context there are two main research questions:

1. Will it be feasible to deliver a brief intervention for depersonalisation symptoms in individuals with current psychotic symptoms?
2. Will such an intervention be acceptable to individuals who experience current psychotic symptoms?

## Methods/design

This study aims to determine the feasibility and acceptability of a brief intervention for depersonalisation symptoms in those with current psychotic symptoms.

The intervention will be evaluated using a single-blinded (researcher blinded) randomised controlled trial (RCT) with a treatment as usual control condition. Participants will be assessed at baseline (T1) and then randomised to either the treatment or control arm. Participants randomised to the treatment arm will be offered six sessions of individual therapy delivered over a maximum of 10 weeks (to allow for non-attendance). All participants will be assessed again at a 10-week post-randomisation follow-up assessment.

### Aims and objectives

The specific aims of the study are to establish:

- The feasibility of:
  - ○ Recruitment, including eligibility rates and acceptance rates and the randomisation process
  - ○ Delivering a brief CBT intervention for DPD, including attendance and completion rates
- The acceptability of the intervention for participants including estimates of satisfaction and treatment adherence

The secondary aim is to establish estimates of standard deviations for outcomes of depersonalisation symptoms to inform sample size calculations for a future trial.

### Participants

We will seek to recruit 30 adults aged 18–70 with current psychotic symptoms and whose depersonalisation symptoms meet threshold for DPD (i.e. over 75 on the Cambridge Depersonalisation Scale (CDS)). We will exclude those with insufficient capacity to provide informed consent; insufficient proficiency in English (spoken and written) to engage in CBT; a primary diagnosis of intellectual disability, head injury, substance misuse or organic cause for psychosis; and those currently engaging in CBT or other psychotherapy.

### Power calculation

As this is a feasibility study and the aim is to provide estimates of key parameters for a future trial rather than to power the current study to detect statistically significant differences, an a priori power calculation was not conducted [32]. Instead, we aim to recruit sufficient participants to provide reasonable estimates of study parameters. Based on the feasibility of recruitment, we aim to recruit 30 participants. Two pieces of work enable estimation of the number of patients we would need to screen in order to obtain 30 participants. Published research with a similar sample [28] suggests that approximately 94 % of individuals diagnosed with a current psychotic disorder will report at least one depersonalisation symptom, and 60 % will experience at least 10 depersonalisation symptoms often. A recently completed research study (Emma Davies, unpublished thesis, 2015) recruiting people with psychosis from the same pools as proposed for this trial suggests that approximately 50 % of participants reporting depersonalisation symptoms met criteria for DPD. In this context, we are likely to need to screen 60 participants (assuming most individuals will report at least one symptom and 50 % will score above our threshold) to obtain our target sample. As it is unlikely that all those contacted via initial letter will respond and/or agree to be screened, we anticipate attempting to contact approximately 100 individuals to be able to screen 60.

### Intervention

The intervention is based on the protocol developed for CBT for DPD [31]. The intervention aims to reduce distress associated with depersonalisation symptoms by altering catastrophic attributions through psycho-education, developing a shared understanding linking depersonalisation symptoms to anxiety and/or past traumas, enhancing coping strategies (including grounding) and cognitive restructuring techniques to modify unhelpful appraisals. It is hypothesised that through reducing distress, in particular, the maintenance cycle associated with depersonalisation will be altered and thus overall depersonalisation symptoms reduced.

The intervention will be delivered, in addition to treatment as usual (see below) over six, 60-min sessions, covering the areas outlined in Table 1, as appropriate and determined by the individual needs of the participant. Sessions will be conducted at outpatient consulting rooms closest to the participant or their home, depending on participant preference and needs.

**Table 1:** Components of CBT intervention

Psycho-education/shared formulation • Psycho-education about depersonalisation • Individualised CBT shared formulations for current pattern of depersonalisation • Assessing factors which influence fluctuations in severity • Rationale for keeping a diary for homework • Example of diary completion Behavioural • Planning and testing impact of environmental/behavioural changes to manipulate and manage depersonalisation symptoms Emotion regulation • Examining the role of emotions associated with depersonalisation • Identifying anxiety/distress management strategies • Psycho-education about grounding strategies and practice of these Cognitive • Identifying unhelpful thoughts about depersonalisation • Cognitive restructuring—reviewing the evidence for and against unhelpful depersonalisation related thoughts Thinking processing • Role of attention in maintaining depersonalisation • Reducing hyper-vigilance/symptom monitoring/checking behaviours • Acceptance and mindfulness approaches to depersonalisation Review and relapse prevention • Summary of what has been learnt from the sessions • Depersonalisation action plan After each session, participants will be given a small ‘homework’ task to practice techniques introduced in the session and to monitor symptoms using a diary	

The therapy will be delivered by SF, a clinical psychologist in training under the supervision of EH, a consultant clinical psychologist and developer of the cognitive model of DPD [20]. In order to ensure the best delivery of the intervention, the therapist (SF) will be trained by EH. Regular supervision through the intervention period of the study will be provided by EH. In addition, to ensure the competence in CBT and fidelity of the intervention, a random selection of 10 % audio recordings of intervention sessions will be rated by EH using a well-established adherence measure of CBT [33] and a measure designed at the start of the study to capture fidelity to the depersonalisation protocol.

### Treatment as usual control condition

For most participants, the treatment as usual will involve regular contact with a care coordinator, medication and regular reviews with a psychiatrist as provided for under the care programme approach (CPA [34]).

### Procedure

Eligible participants will be recruited from a secondary care mental health trust in South London (SLaM) and will include community mental health teams, psychological therapies services and research registers.

Potential participants identified from the sources above will be sent a letter of invitation and study information in the post. The letter will provide detail of how to contact the researcher should they be interested. If after 1 week, there has been no contact from the participant, the first author will telephone them, answer any questions they may have about the study and offer an opportunity to be screened for eligibility. This screening interview ask questions about current experiences of hallucinations and paranoia or other delusions and will determine whether they are likely to meet criteria for diagnosis of DPD using the Cambridge Depersonalisation Scale [35]. Participants passing this initial screen will then be invited to a face-to-face interview with a researcher and be invited to provide informed consent to participate in the study before participating in the baseline assessment

**Fig. 1:**
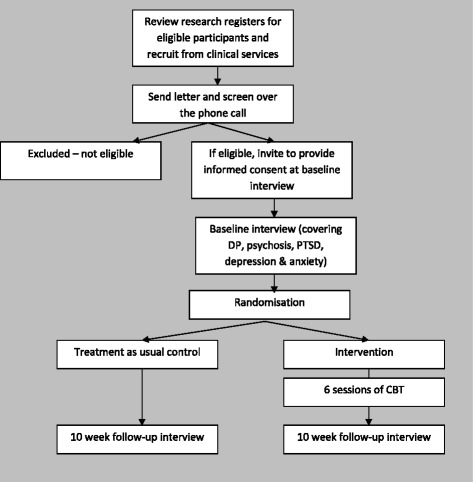
Trial flowchart

All assessments will be conducted by an independent research assistant, trained in the administration of measures and who will remain blind to treatment allocation; maintenance of blinding will be collected at outcome assessment. After the completion of the follow-up interview, researchers will be unblinded and will re-contact those who received the intervention to assess satisfaction with and acceptability of the intervention. See Fig. 1 for the trial flowchart.

### Randomisation

An online randomisation service will be used to allocate participants to either the intervention group or control. Randomisation will use randomly permuted blocks to ensure equal allocation to each group. After the baseline interview, the first author will enter the participant’s details into the online service and will receive an automatic email which details the allocation of the participant. The first author will then contact the participant to alert them of their allocation and, if the CBT/active intervention group, will arrange first therapy session. The RA will be kept blinded to the allocation to reduce bias.

### Data collection

The primary outcomes of this trial are the estimates of feasibility and acceptability.

To establish the feasibility of conducting a future trial, the following data will be collected throughout the recruitment and intervention process:

- Referral (number of participants referred to the study)
- Eligibility rates (number of referred and approached participants who meet study entrance criteria)
- Acceptance rates (number of participants consenting to the study) and reasons for study refusals
- Participant attendance rates at sessions and duration of intervention (i.e. number of weeks taken to attend six sessions)
- Data attrition (proportion of outcome data obtained)
- Feasibility of randomisation process (maintenance of researcher blindness to treatment allocation)
- Therapist competence, CBT fidelity and CBT for DPD

To establish the acceptability of the intervention, in addition to the data above, attrition rates (number of treatment sessions completed by participants, number of therapy drop outs (i.e. completing two or fewer sessions) and percent of homework tasks completed) will be collected throughout the intervention.

After the follow-up interview is completed and scored, researchers will be unblinded and all intervention participants will be interviewed about their impressions of the intervention. The interview will collect data on their impressions of and satisfaction with the intervention using questions based on the Satisfaction of Therapy Questionnaire [36], altered to capture aspects of improvement/satisfaction related to depersonalisation symptoms. Participants will be asked to rate on a five-point Likert scales:

- ○ Their expectations and actual progress made on dealing with depersonalisation
- ○ Their level of satisfaction with the therapy, therapist and tasks between therapy
- ○ The extent to which they gained new skills and knowledge during the intervention
- ○ Their relationship to the therapist including therapist competence, sympathy, caring nature and supportiveness

There will be four additional open questions to determine the aspects of the intervention the participants found most and least helpful, their views on whether the intervention met their expectations overall and an opportunity to make any other comments.

### Clinical data

Clinical and demographic data will be collected at baseline interview and will include sex, age, ethnicity, marital status, education, employment status, medication use, age of onset of both DP/DR and psychotic symptoms, current clinical diagnosis and past experience of cognitive behavioural therapy or other psychotherapeutic approaches. Data will be collected at baseline assessment and at an outcome interview at 10 weeks.

Secondary, clinical outcome data will be collected to estimate key parameters to inform future trial design. Outcomes include depersonalisation, psychotic, depression and anxiety symptoms, as well as screening for post-traumatic stress disorder.

- Cambridge Depersonalisation Scale (CDS; [35]). The CDS is a 29-item scale that measures the severity of trait depersonalisation symptoms over the preceding 6 months. For each item, frequency (Likert scale 0 = never to 4 = all the time) and duration (Likert scale 1 = few seconds to 6 = more than a week) are collected; each item maximum is therefore 10. A total scale score is the sum of each item, with a maximum of 290. Scores greater than 70 have been shown to reliably predict a clinical diagnosis of DPD using DSM criteria. In order to measure change, the wording of the trait CDS will be changed to measure the severity of DP/DR symptoms *over the preceding month*. The level of distress, preoccupation, impairment and understanding of depersonalisation symptoms will also be collected.
- The Psychotic Symptom Rating Scale (PSYRATS; [37]). The PSYRATS will be used to monitor changes in psychotic symptomatology between baseline assessment and outcome interview. The PSYRATS consists of two subscales measuring the presence and typology, beliefs/conviction, distress and disruption associated with auditory hallucinations and delusions. The auditory hallucination (AH) subscale has 11 items and the delusions (DELs) subscale has six items. All items are scored between 0 and 4. For example, for item 1 in the AH scale, 0 = voices are not present to 4 = voices are present continuously… The maximum score for the AH and DELs subscales are 44 and 24, respectively.
- Beck Depression Inventory (BDI; [38]). The BDI-II is a 21-item self-report scale, rated on a 4-point Likert scale (0 = symptom not present to 3 = symptom present with significant distress/impairment) measuring common symptoms of depression. Total scores range from 0 to 63. Total scores of less than 13 indicate minimal depression, scores 14–19 indicate mild depression, scores 20–28 indicated moderate depression and scores above 29 indicate severe depression.
- Beck Anxiety Inventory (BAI; [39]). The BAI is a 21-item self-report scale with the same scoring. Total scores are interpreted as follows: 0–9 indicates minimal anxiety; 10–16 indicates mild anxiety; 17–29 indicates moderate anxiety; and 30–63 indicates severe anxiety.
- Post-traumatic Diagnosis Scale (PDS; [40]. The PDS has 49 items including a checklist of potentially traumatising events and an indication of the distress, intrusive thoughts, avoidance and hyperarousal in the last month. There is a total score ranging from 0 to 51 with 1–10 considered ‘mild’, 11–20 ‘moderate’, 21–35 moderate to severe and greater than 36 severe.
- Structured clinical interview for DSM-IV dissociative disorders (SCID-D) [41]. It includes nine items addressing the presence and frequency of common depersonalisation symptoms, the duration and frequency of the most severe instance of depersonalisation, functional impairment, distress and exclusionary criteria such as not the result of drugs, organic issues and does not occur exclusively in the context of other psychiatric condition such as psychosis.

### Analyses

As this is a feasibility study, the analyses will be primarily descriptive aiming to provide estimates of feasibility parameters and to inform power calculations for a future trial. Descriptions of continuous data, including clinical data and sample characteristics, will be provided using mean, SD, median and IQR. Frequencies and proportions will be used to analyse categorical variables.

Feasibility of trial procedures will be assessed using proportions and their estimated 95 % confidence intervals (CIs) for rates of the following: referral (number of referrals divided by total approached); eligibility (number of eligible participants divided by number screened and number approached); acceptance (number screened divided by number approached and number consented divided by number approached); attendance (average number of treatment sessions attended and average number of weeks taken to complete intervention); data attrition (proportion of outcome data obtained); and maintenance of blinding (incidences of unblinding of researcher divided by number of follow-up assessments). Therapist competence will be presented as proportion with estimated 95 % confidence intervals for the total score divided by applicable items on the CTRS and DPD fidelity measure. Acceptability of trial procedures will be assessed using proportions and their estimated 95 % confidence intervals for rates of: attrition (proportion of treatment sessions completed and of homework tasks completed), expectations and actual progress (proportion rating at each point on Likert scale) and satisfaction with therapy, therapist and tasks (proportion rating at each point on Likert scale).

Population variances will be determined using the upper 80th nonparametric bootstrap percentile of confidence intervals around the estimates [42].

### Adverse events

We do not anticipate any serious adverse events as a result of this psychological intervention, but all adverse events will be collected, discussed in supervision and reported to regulatory authorities as required.

## Discussion

This paper presents the protocol for a study to assess the feasibility and acceptability of a brief cognitive behavioural therapy intervention for individuals who have depersonalisation symptoms in the context of psychotic symptoms. The intervention, based on that developed for DPD, will focus on providing an individual cognitive formulation and explanation of depersonalisation experiences and developing behavioural, cognitive, emotional regulation and thinking processing changes or strategies to decrease the associated distress. The findings from this study will help estimate the key parameters for a future trial.

### Trial status

Recruitment to the trial is underway and is due to be completed in March 2016. The first participant was randomised in June 2015.

## Abbreviations

BAI, Beck Anxiety Inventory; BDI, Beck Depression Inventory; CBT, cognitive behavioural therapy; CDS, Cambridge Depersonalisation Scale; CI, confidence intervals; DPD, depersonalisation disorder; PDS, Post-traumatic Diagnosis Scale; PSYRATS, The Psychotic Symptom Rating Scale; PTSD, post-traumatic stress disorder; SCID-D, structured clinical interview for DSM-IV dissociative disorders; UK, United Kingdom; USA, United States of America
